# Comparison of the Effect of Two Different Handling Conditions at Slaughter in Saliva Analytes in Pigs

**DOI:** 10.3390/metabo14040234

**Published:** 2024-04-18

**Authors:** María Botía, Damián Escribano, Alba Ortín-Bustillo, María J. López-Martínez, Pablo Fuentes, Francisco J. Jiménez-Caparrós, Juan L. Hernández-Gómez, Antonio Avellaneda, José J. Cerón, Camila P. Rubio, Asta Tvarijonaviciute, Silvia Martínez-Subiela, Marina López-Arjona, Fernando Tecles

**Affiliations:** 1 Interdisciplinary Laboratory of Clinical Analysis (Interlab-UMU), Veterinary School, Regional Campus of International Excellence ‘Campus Mare Nostrum’, University of Murcia, Campus de Espinardo s/n, 30100 Espinardo, Spain; maria.botiag@um.es (M.B.); det20165@um.es (D.E.); alba.ortinb@um.es (A.O.-B.); mariajose.lopez28@um.es (M.J.L.-M.); jjceron@um.es (J.J.C.); camila.peres@um.es (C.P.R.); asta@um.es (A.T.); silviams@um.es (S.M.-S.); ftecles@um.es (F.T.); 2 Department of Animal Production, Regional Campus of International Excellence ‘Campus Mare Nostrum’, University of Murcia, Campus de Espinardo s/n, 30100 Espinardo, Spain; 3 Cátedra de Seguridad y Sostenibilidad Alimentaria Grupo Fuertes-Universidad de Murcia, 30003 Murcia, Spain; pablo.fuentes@cefusa.com (P.F.); franciscojavier.jimenez@elpozo.com (F.J.J.-C.); juanluis.hernandez@elpozo.com (J.L.H.-G.); antonio.avellaneda@elpozo.com (A.A.); 4 Department of Animal and Food Science, Universitat Autònoma de Barcelona, 08193 Cerdanyola de Vallés, Spain

**Keywords:** biomarkers, porcine, saliva, slaughterhouse, welfare

## Abstract

In this report, different handling conditions at slaughterhouse were studied to assess changes in salivary biomarkers. For this purpose, finishing pigs were divided into two groups, one in which handling was improved to minimize stress (Group A, *n* = 24, transported and stabled at the slaughterhouse at low density without mixing with unfamiliar animals throughout the whole process) and another one in which animals had a more stressful handling process (Group B, *n* = 24, transported and stabled at high density with unfamiliar animals). Saliva samples were taken the day before transport to the slaughterhouse at 8:00 a.m. (B0) and 12:00 a.m. (B4), and the day of slaughter just after unloading animals at the slaughterhouse at approximately 8:00 a.m. (S0) and after 4 h of lairage at approximately 12:00 a.m. (S4). Group B showed significantly higher cortisol, total esterase activity, oxytocin, adenosine deaminase and haptoglobin levels than the Group A at both S0 and S4 sampling times, and higher levels of calprotectin and creatine kinase at S4 sampling time. This report indicates that differences in the way in which the pigs are handled at the slaughterhouse can lead to changes in salivary biomarkers and opens the possibility of the use of biomarker at slaughter to monitor handling conditions.

## 1. Introduction

In the swine industry, the pre-slaughter period covers a period of around 24 h before animals are slaughtered. This time is especially stressful for the animals [1,2]. Several factors can induce this stress, including the previous feed deprivation, regrouping and housing in pick-up facilities prior to loading animals onto the truck, the loading procedure, transport (including length, stacking density and bad weather conditions), unloading at the slaughterhouse, and regrouping and housing in the lairage with unfamiliar animals, among others [3,4,5]. Fatigue accumulates in the animals as long as all these stressors act, which can lead to a lesser quality of the meat after slaughter. Improving handling at the pre-slaughter period would be of high importance, not only for assuring animal welfare, but also for commercial purposes since the better stressor factors are controlled, the better meat quality is obtained [1,2].

The Council Regulation (EC) No. 1099/2009 (https://eur-lex.europa.eu/eli/reg/2009/1099/oj, [last accessed on 1 May 2023]) indicates that those slaughterhouses that slaughter over 1000 animals per year should document animal welfare. Several tools can be used for this purpose, including animal behavior, environmental data, or physiological variables [6]. The Welfare Quality^®^ protocol (WQ^®^, 2009; Welfare Quality^®^ (2009). Welfare Quality^®^ Assessment protocol for pigs. Lelystad, Netherlands: Welfare Quality^®^ Consortium978-90-78240-05-1; http://www.welfarequalitynetwork.net/en-us/reports/assessment-protocols, [last accessed in 1 March 2024]) collects data regarding environment, health status and behavior of the animals and it has been used to assess welfare at farm level and also at the slaughterhouse in the period comprised from unloading until slaughter [7,8]. In this context, the use of biomarkers could be an additional complementary tool for welfare assessment, since they have the potential for detecting the stress that the animal suffers. This is the case of blood biomarkers such as cortisol, creatine kinase (CK), lactate dehydrogenase (LDH), total protein (TP), albumin [9,10] or lactate [11]. However, blood collection in pigs is a highly stressful procedure since it requires animal restriction, which could affect the results of the stress related biomarkers.

In contrast to blood sampling, saliva can be obtained safely, by non-trained staff, allowing repeated samplings over time without producing evident stress to the animals. In addition, several biomarkers that can provide information about stress, immunity, inflammation, and redox status can be successfully measured in this fluid [12]. Saliva has been used for assessing welfare at slaughterhouse level. For example, some studies have found that salivary cortisol concentrations were increased after transportation to the slaughterhouse and values trend to decrease during lairage [13,14]. Interestingly, salivary cortisol can increase again if lairage length is too long, since other factors such as food deprivation, background noise, a high animal density, mixing with unfamiliar animals or recent mixing of genders are possible stressors contributing to raised cortisol [13]. Other salivary biomarkers such as oxytocin, alpha-amylase (sAA), total esterase (TEA), butyrylcholinesterase (BChE) and LDH have shown changes at slaughterhouses when compared with values recorded before transportation [15].

Based on the previous results, we hypothesize that saliva biomarkers can be potentially useful for assessing different handling conditions of animals at slaughterhouse level, being a potential tool to differentiate between conditions producing different degrees of stress. Thus, the aim of this research was to study how two different handling conditions from loading until slaughter can affect salivary biomarker levels. For this, two groups of animals, one in which al handling was performed trying to avoid and/or reduce the main stressors, and another handled the inclusion of additional stressors, were sampled for saliva and a profile of biomarkers was studied.

## 2. Materials and Methods

### 2.1. Animals and Experimental Procedure

A total of 48 pigs [(*Sus scrofa domesticus*) (Large White × Duroc) (24 male × 24 female)] at the end-fattening period (5–6 months of age and an approximate body weight of 116 kg) were included in this study. The animals were housed in a farm in southern Spain in groups of 14 animals with a minimum space of 0.65 m^2^ per animal, and they were given ad libitum access to a balanced diet and water. The experiment took place during February of 2022. At the end of the fattening period, all pigs were transported the same day to a commercial slaughterhouse. The transport was according to the recommendations described in Directive 2001/88/EC, 2001 and Directive 2001/93/EC, 2001.

The animals were divided into two different groups:

(A) Group A (improved handling group, 12 males, 12 females). The animals were loaded into the truck and transported for 15 km to the slaughterhouse in groups of 10 animals (1.25 m^2^ per animal), unloaded on arrival at the slaughterhouse and placed in a lairage area with free access to water. In order to minimize stress, those animals were the last to be loaded onto the truck, the first to be unloaded once at the slaughterhouse, and they were not mixed with unfamiliar ones in order to avoid the establishment of new hierarchical relationships with 1.25 m^2^ per animal.
(B) Group B (stressful handling group, 12 males, 12 females). Animals from this group were loaded into the same truck and transported for the same length, but they were mixed with other animals at 0.55 m^2^ per animal. In order to increase stress level, they were the first animals to be loaded onto the truck and the last to be unloaded from it, thus the processing time was increased. After arrival at the slaughterhouse, the animals were mixed with unfamiliar animals using higher density (0.38 m^2^ per animal) with free access to water.

After four hours of lairage, all animals were stunned with a CO_2_ concentration over 80% and slaughtered according to Directive (CE) 1099/2009, published in OJEU-L-2009-82167.

### 2.2. Sampling Procedure

Saliva samples were collected on two consecutive days. The day just before transport, saliva was collected at farm level at approximately 8:00 a.m. (B0) and then four hours later (B4). On the day of slaughter, samples were taken immediately after unloading animals at the slaughterhouse at approximately 8:00 a.m. (S0), and also after lairage of four hours at approximately 12:00 a.m. (S4). For sampling, the pigs were individually allowed to chew a 5 × 2 × 2 cm volume piece of a polypropylene sponge (Esponja Marina, La Griega E. Koronis, Madrid, Spain), which was clipped to a metal rod so the researcher can handle the sponge to be bitten by only one animal at a time. After approximately 1 min, and after the researcher checked that the sponge was thoroughly moistened, the sponges were placed into Salivette tubes (Sarstedt, Aktiengesellschaft & Co. D-51588 Nümbrecht, Germany) and maintained refrigerated in a box with cold storage accumulators until arrival at the laboratory. Once at the laboratory, tubes were centrifuged at 3000× *g* and 4 °C for 10 min. Supernatants were collected and stored at −80 °C until analysis.

### 2.3. Salivary Biomarkers Measurement

The biomarkers measured in this report were previously validated in porcine saliva by our research group [16,17]. The following biomarkers were measured (additional details are provided at Supplementary Material).

#### 2.3.1. Stress Biomarkers 

Cortisol was measured by a commercial solid-phase, competitive chemiluminescence enzyme immunoassay (Cortisol, REF LKC01, Siemens Health Diagnostics, Deerfield, IL, USA). For sAA measurement, a colorimetric commercial kit (Alpha-Amylase, Beckman Coulter Inc., Fullerton, CA, USA) was used. Salivary TEA was measured using 4-nitrophenyl acetate as substrate (Sigma-Aldrich Co, St. Louis, MO, USA). Salivary BChE was analyzed by an automated spectrophotometric assay using butyrylthiocholine iodide (BTCI, Sigma-Aldrich Co., St. Louis, MO, USA) as substrate. Oxytocin was determined with an AlphaLISA method (PerkinElmer, Waltham, MA, USA). 

#### 2.3.2. Immune System and Muscle Biomarkers

Adenosine deaminase (ADA) was measured by a commercially available spectrophotometric assay (Adenosine Deaminase assay kit, Diazyme Laboratories, Poway, CA, USA). Haptoglobin (Hp) was measured by an in-house assay based on AlphaLISA method. Calprotectin was determined by the BÜHLMANN fCal Turbo^®^ assay (BÜHLMANN, Laboratories AG, Schönenbuch, Switzerland). CK was measured using a commercial kit from Beckman (Beckman Coulter Inc., Fullerton, CA, USA). 

The analyses were performed in an Olympus AU600 biochemistry autoanalyzer (Olympus AU600, Olympus Diagnostica GmbH, Ennis, Ireland), except cortisol, Hp and oxytocin, which were measured with the Immulyte system and/or a 96-well plate reader (PerkinElmer, Inc., Waltham, MA, USA).

### 2.4. Statistical Analysis

The Shapiro–Wilk test was performed to evaluate the data distribution; since they did not follow a normal distribution, data were naturally log-transformed prior to analysis. A mixed lineal model was performed in which time and group were considered as fixed factors, and the individual as a random factor. Different covariance structures were tested (autoregressive first order, compound symmetry and unstructured) and that providing lower Akaike’s criteria was selected. The Bonferroni post-hoc test was used for B0 vs. S0 and B4 vs. S4 pairwise comparisons. Graphics were performed using a spreadsheet (Excel 2000, Microsoft Corporation, Redmond, Washington, DC, USA) and the statistical package Graph Pad (GraphPad Prism, version 6 for Windows, Graph Pad Software Inc., San Diego, CA, USA), and statistical analyses were performed with the statistical package SPSS (IBM SPSS Statistics for Windows, Version 28.0. IBM Corp., Armonk, NY, USA). Statistical significance was set to α = 0.05.

## 3. Results

### 3.1. Stress Biomarkers

**Figure 1:**
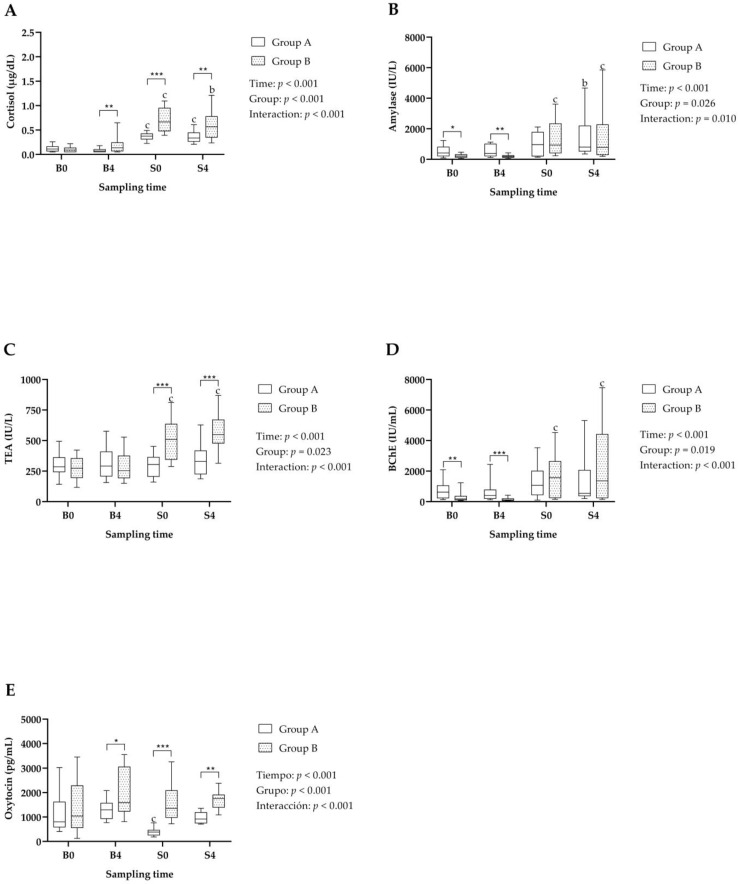
Salivary stress biomarker levels [(**A**) Cortisol; (**B**) Amylase activity; (**C**) Total esterase activity (TEA); (**D**) Butyrylcholinesterase activity (BChE); (**E**) Oxytocin] in the improved handling group (Group A) and stressful handling group (Group B), obtained the day before transport to slaughterhouse 8:00 a.m. (B0) and 12:00 a.m. (B4), and the day of slaughter just after unloading animals at the slaughterhouse at approximately 8:00 a.m. (S0) and after 4 h of lairage at approximately 12:00 a.m. (S4). Statistical analysis: letters indicate significant differences within each group between sampling points at the same hour of the day (b: *p* < 0.01; c: *p* < 0.001); asterisks indicate differences between groups at a given sampling point (*: *p* < 0.05; **: *p* < 0.01; ***: *p* < 0.001).

Results of the stress biomarkers are shown in Figure 1. Significant Time, Group and Time*Group interaction effects were observed in cortisol (Figure 1A), sAA (Figure 1B), TEA (Figure 1C), BChE (Figure 1D) and oxytocin (Figure 1E). Both groups A and B showed increases in cortisol at S0 and S4 compared with the values obtained at B0 and B4, respectively. In addition, differences were observed depending on the group, since Group B had values of cortisol and TEA at S0 and S4 significantly higher than Group A. In this line, only Group B showed increases in BChE at S0 and S4 compared with B0 and B4, respectively. 

In contrast to those previous biomarkers, oxytocin showed a significant decrease in Group A at S0 compared with B0. No significant changes were observed in Group B, which also had significantly higher values than Group A at S0 and S4. 

### 3.2. Immune System and Muscle Biomarkers

**Figure 2:**
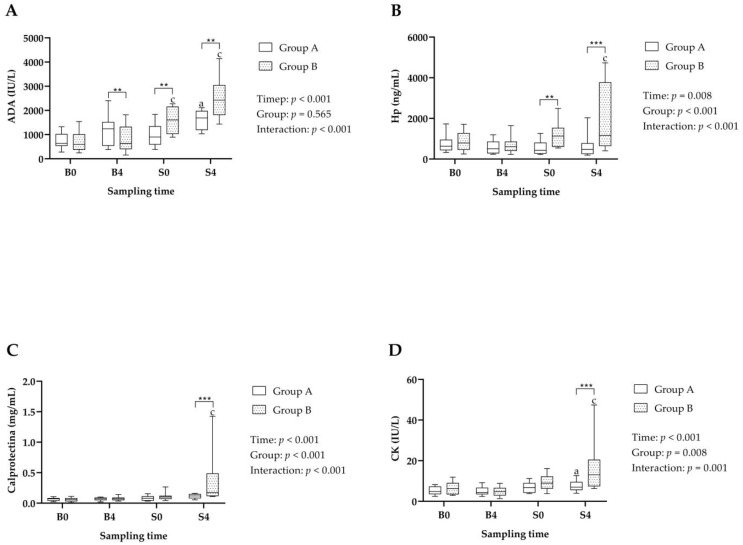
Salivary biomarkers of immunity [(**A**) Adenosine deaminase, ADA; (**B**) Haptoglobin, Hp; (**C**) Calprotectin] and muscle [(**D**) Creatine kinase, CK] levels in the improved handling group (Group A) and stressful handling group (Group B), obtained the day before transport to the slaughterhouse 8:00 a.m. (B0) and 12:00 a.m. (B4), and the day of slaughter just after unloading animals at the slaughterhouse at approximately 8:00 a.m. (S0) and after 4 h of lairage at approximately 12:00 a.m. (S4). Statistical analysis: letters indicate significant differences within each group between sampling points at the same hour of the day (a: *p* < 0.05; c: *p* < 0.001); asterisks indicate differences between groups at a given sampling point (**: *p* < 0.01; ***: *p* < 0.001).

Results of the immune system and muscle biomarkers appear in Figure 2. The Time*Group interaction was significant in ADA (Figure 2A), which showed significant increases in Group B at S0 and S4 compared to B0 and B4, respectively; whereas Group A showed only a significant increase at S4 compared to B4. In addition, the changes in ADA were of higher magnitude in Group B, whose values were significantly higher at both slaughterhouse time points than in Group A.

Significant Time, Group and Time*Group interactions were observed in Hp (Figure 2B) and calprotectin (Figure 2C). Both biomarkers showed a significant increase in Group B after 4 h of lairage in the slaughterhouse (S4) compared with the values observed at farm at the same time point (B4). In addition, differences were observed depending on the group, with Hp being significantly higher in Group B than in Group A at S0 and S4, and calprotectin at S4. 

Significant Time, Group and Time*Group interactions were observed in CK (Figure 2D). It showed a significant increase in both groups A and B after 4 h of lairage in the slaughterhouse compared with the values observed at the farm in the same time point (B4). When the different groups were compared, CK at S4 was significantly higher in Group B than in Group A.

## 4. Discussion

The stress at the pre-slaughter period can be induced by several factors, and its adequate control can improve the welfare of the animals and quality of the final product [1,18]. In this report, saliva samples were used for evaluating stress and welfare, which have the advantages of being obtained safely, by non-invasive procedures executed by animal-care personal without the need of the assistance by veterinarians. Additionally, saliva collection allows repeated samplings over time without producing evident stress to the animals [12], in contrast to blood which usually requires animals to be restrained, producing a more stressful situation. The main purpose of this report was to evaluate how two different handling conditions with different degrees of stressful factors could have an effect on salivary analytes. A comprehensive panel of analytes was used, including not only stress biomarkers but also biomarkers of inflammation and immunity and muscle damage.

The different handling procedures were applied from transport to the slaughterhouse until animals were slaughtered. Because the length of transport an important stressor [19,20] the overall duration of the transport was increased in Group B since these animals were the first to be loaded onto the truck and the last ones to be unloaded. In addition, Group B was mixed with unfamiliar animals and transported with a higher animal density, a situation that increases stress in short-term transport [1,20]. Once at the slaughterhouse, Group A was not mixed and stabled at low density, whereas Group B was mixed with unfamiliar animals, which led to an aggressive behavior since a new hierarchy had to be established [21], and stocking density was increased. Due to all these factors, we expected a higher stress level in Group B compared with Group A. 

In our conditions, when the salivary biomarkers were compared between groups, cortisol, oxytocin, TEA, ADA and Hp showed significant differences between the animals handled in different conditions at both S0 and S4 sampling times, and calprotectin and CK after 4 h of lairage at the slaughterhouse (S4 sampling time). In all these cases, Group B showed higher values than Group A. This would indicate a higher stress level in these animals and that the transport and lairage period at the slaughterhouse were strong enough stressors to induce changes in several salivary biomarkers after transport and 4 h of lairage at the slaughterhouse.

Cortisol was increased in both groups at the slaughterhouse compared with the values observed at the farm, which is in line with previous studies after transport and lairage at slaughterhouses [13,15,22]. The hypothalamic–pituitary–adrenal axis (HPA) responses against stress-releasing glucocorticoids [23], with cortisol being the main glucocorticoid in pigs and the most widely used biomarker to detect stress in this species [24]. Salivary cortisol reflects the circulant active fraction [25], so it is widely used for assessing stress in pigs. In spite of its high sensitivity in detecting stress, since cortisol was increased in both groups of animals compared with the values at farm, salivary cortisol was significantly higher in Group B than in Group A at both times at the slaughterhouse, thus indicating that it could be a valuable biomarker for the detection of different degrees of stress. 

Another biomarker of stress that showed significant changes between the different handling systems was TEA. It is a marker of the sympathetic–adrenal–medullary (SAM) system that prepares the animal to deal with stressful situations [26]. In this report, increases compared with values at the farm were seen after transport and 4 h of lairage in TEA only in Group B, indicating that this group suffered a higher stress than Group A. 

Oxytocin has been used as a biomarker of positive situations [27], since in previous studies it increases in pigs after being stroked [28] or after ejaculation [29], whereas it is decreased in pigs with tail-biting lesions [30]. Oxytocin has been reported to decrease in pigs after transport and 4 h of lairage at slaughterhouses under routine conditions [15], similarly as observed in this research in Group A. In contrast, Group B, which suffered a higher stress, did not show any change in the salivary oxytocin concentration compared with farm concentrations. The relationship of oxytocin with stress seems to be complex, since oxytocin can modulate differently the stress response depending on the social situation of the individual. It has been proven in rats that stress due to shaking of the animals can increase oxytocin in the hypothalamic paraventricular nucleus [31]. In addition, oxytocin increases its circulating values due to social anxiety and stress [32], for example due to bad social relationships [33], social isolation [34], or chronic social stressors [35]. This increase in oxytocin due to social stress seems to be related to cortisol release, contributing to downregulation of the stress response [36]. Since Group B was integrated with unfamiliar animals housed in high-density conditions, and therefore suffered a higher social stress, a protective role of oxytocin could be postulated. This would explain why oxytocin did not decrease in Group B compared with Group A—which was composed of animals that were not mixed and suffered less social stress.

Salivary immune system biomarkers were measured in this study, such as ADA, Hp and calprotectin, since the presence of stress affects the immune system response through the release of catecholamines by the SAM, which lead to the induction of pro-inflammatory cytokines such as interleukin-6 inducing the production of acute phase proteins by the liver [37]. In our report, ADA and Hp were higher at the slaughterhouse in Group B than in Group A, thus indicating a response due to the higher stress that the animals from Group B suffered. In fact, ADA has been proven to be increased in pigs suffering distress due to pathologic conditions [38]. Regarding Hp, the results provided by literature are contradictory, since increased levels have been reported in serum after 20 min of transport and 3 h of lairage [39], whereas a 45 min transport or social isolation did not produce changes in salivary Hp levels [40]. A possible explanation for those contradictory results could be the different methods used for Hp determination. Calprotectin was significantly increased in Group B after 4 h of lairage at the slaughterhouse. Although the relationship between stress and calprotectin has not been fully studied, some studies in patients with inflammatory bowel disease have associated the presence of higher levels of stress with increased fecal calprotectin [41], and also in a study involving preterm infants from neonatal intensive care units found a relationship between cumulative pain/stress during hospitalization and fecal calprotectin concentrations [42]. 

The marker of muscle damage CK was increased in both groups after 4 h of lairage, and this increase was higher in Group B. CK has been reported to be increased in the serum of animals after transport and arrival at the slaughterhouse; and authors reported increased CK values after lairage of 5 h [43] or 24 h [9]. The reason for the increase obtained in our report could be a higher incidence of muscle damage when animals are in lairage [43]. It is interesting to note that the recovery of serum CK levels to baseline could take up to 120 h, according to previous studies in humans [44].

The results observed in this study could have important practical applications since they can lay the foundations of the use of salivary biomarkers as a tool for stress evaluation at the slaughterhouse. These results demonstrate that saliva can be useful for the discrimination between different handling conditions at the slaughterhouse and can also help to select and monitor the most appropriate conditions to reduce stress. For this purpose, further studies involving a large number of animals and also more slaughterhouses with different environmental and housing conditions should be performed.

This study has several limitations. The first one is the limited number of animals included and that the experiment has been performed only once and without replications which may limit the generalizability of the findings. The experimental conditions should be replicated in a higher number of individuals, that can lead to the development prediction tools such as cut-off point calculations, regression analyses or algorithm construction. It is also important to take into account that the results in this study were obtained under specific conditions and could vary under other different conditions or ways of handling. In addition, the way in which animals respond to stress could depend on factors such as breed, age and even the season of the year [45], which were not considered in this report. The secretion of cortisol follows a diurnal pattern [46], which needs to be taken into consideration when measuring cortisol. In our report, this effect was minimized since each value observed at the slaughterhouse was compared with values obtained in the farm at the same hour of the day. In our study, time*gender and group*gender interactions were not significant and therefore we decided to perform the statistical study taking all the animals together without sex differentiation. However, gender could be a source of variation for salivary analytes [47] and additional studies with a larger number of animals should be also considered to detect possible differences in the gender response to stress at the slaughterhouse. Finally, it is also important to consider that the biomarker levels could change according to the environmental conditions or health status of the animals. However, in this report all animals came from the same farm so environmental conditions were similar for all of them. In addition, pigs in this study did not show evident signs of illnesses, and no evidence of lesions were recorded after post-mortem examinations. For those reasons, overall this study should be considered as a pilot one that indicates the potential of salivary biomarkers to differentiate between different handling conditions at slaughterhouses.

## 5. Conclusions

Changes in salivary biomarkers are observed for different handling conditions both in transport and at the slaughterhouse. The stress biomarkers cortisol, TEA and oxytocin, and the immunity biomarkers ADA and Hp, showed differences between groups at arrival; and all these biomarkers, as well as a calprotectin and CK, also showed changes between groups after 4 h at the slaughterhouse. Thus, the use of salivary biomarkers could be a potentially useful tool to assess the effect of different handling situations and reduce the presence of stress in pigs at slaughterhouse level. This can contribute to better management and control of those situations that produce stress at this productive level. Further studies should be made in order to gain more knowledge about the potential use of salivary biomarkers in routine to evaluate the welfare of animals at the slaughterhouse.

## Data Availability

The raw data supporting the conclusions of this article will be made available by the authors on request.

## Supplementary Materials

The following supporting information can be downloaded at: https://www.mdpi.com/article/10.3390/metabo14040234/s1, Table S1: Assays used for the measurement of salivary stress biomarkers. Table S2: Assays used for the measurement of salivary immunity and muscle biomarkers.
