# Supplementary material for: Comparison of the Effect of Two Different Handling Conditions at Slaughter in Saliva Analytes in Pigs
Source: Metabolites. 2024 Apr 18;14(4):234. doi: 10.3390/metabo14040234 (PMC11052431; doi:10.3390/metabo14040234)
Supplement: Supplementary file 1 [file metabolites-14-00234-s001.zip › metabolites-2944337-supplementary.pdf]

Supplementary material

Supplementary Table S1. Assays used for the measurement of salivary stress biomarkers .

Analytical method	Reference	Precision	Accuracy	Limit of detection
Cortisol	Siemens Health Diagnostics, Deerfield, IL.	Within-run: 2.9-11.6%<br>Between-run: 4.3-15.6%	R^2 > 0.995<br>Recovery 91.9%	0.016 µg/dL
sAA	Alpha-Amylase, Beckman Coulter Inc.	Within-run: 0.9-2.1%<br>Between-run: 3.6-9.2%	R^2 > 0.998	11.65 IU/L
TEA	Home-made method using 4-nitrophenyl acetate (Sigma-Aldrich Co, St Louis, Mo, USA) as substrate	Within-run: 1.78-2.57%<br>Between-run: 1.95-3.72%	R^2 > 0.995	2.06 IU/L
BChE	Home-made method using butyrylthiocholine iodide (Sigma-Aldrich Co, St Louis, Mo, USA) as substrate	Within-run: 2.57-4.59%<br>Between-run: 3.94-4.94%	R^2 > 0.998	11.26 IU/mL
Oxytocin	Home-made method using AlphaLISA (PerkinElmer, MA, USA) technology	Within-run: 5.02-6.07%<br>Between-run: 4.6-17.7%	R^2 > 0.959<br>Recovery 104%	112.95 pg/mL

sAA: salivary Alpha-amylase; TEA: Total esterase activity; BChE: butyrylcholinesterase; R^2: linearity under dilution coefficient of determination

Supplementary Table S2. Assays used for the measurement of salivary immunity and muscle biomarkers.

Analytical method	Reference	Precision	Accuracy	Limit of detection
ADA	Adenosine Deaminase assay kit, Diazyme Laboratories, Poway, CA, USA	Within-run: 6.2-8.3%<br>Between-run: 3.9-4.3%	R^2 > 0.999<br>Recovery 93-108%	0.07 IU/L
Hp	Home-made method using AlphaLISA (PerkinElmer, MA, USA) technology	<15%	-	-
Calprotectin	BÜHLMANN fCal Turbo® assay (BÜHLMANN, Laboratories AG, Switzerland)	Within-run: 2.14-4.86%<br>Between-run: 4.26-6.23%	R^2 > 0.946<br>Recovery 110-116.7%	0.01 mg/L
CK	Beckman Coulter Inc., Fullerton, CA, USA	<15%	R^2 > 0.99 (internal data)	0.7 IU/L<br>(internal data)

ADA: adenosine deaminase; Hp: haptoglobin; CK: creatine kinase; R^2: linearity under dilution coefficient of determination
